# Half forehead reconstruction with a single rotational scalp flap for dermatofibrosarcoma protuberans treatment

**DOI:** 10.1186/1477-7819-10-78

**Published:** 2012-05-06

**Authors:** Stefano Mori, Gianluca Di Monta, Ugo Marone, Maria Grazia Chiofalo, Corrado Caracò

**Affiliations:** 1 Department of Surgery “Melanoma - Soft Tissues - Head & Neck - Skin Cancers”, National Cancer Institute, Via Mariano Semmola, 80131, Naples, Italy

**Keywords:** Dermatofibrosarcoma protuberans, Forehead defects, Scalp flap, Local recurrence

## Abstract

**Background:**

Dermatofibrosarcoma protuberans (DFSP) is a soft tissue neoplasm of intermediate to low-grade malignancy. Although metastasis rarely occurs, DFSP has a locally aggressive behavior with a high recurrence rate. In the head and neck area, resection involving a wide margin of healthy tissue can be difficult because of functional and cosmetic considerations. We describe a novel reconstructive method for half forehead defects with an innovative single local wide scalp flap following excision of DFSP with a 3 cm margin of healthy tissue.

**Methods:**

Two patients underwent wide resection of forehead DFSP and reconstruction with a single rotational scalp flap. The scalp flap blood supply was provided from three main vessels: the superficial temporal artery, occipital artery and posterior auricular artery.

**Results:**

No early or late complications were observed in either patient with no local recurrence after 18 months of follow-up. The donor area could be closed primarily in both cases and the flaps survived completely.

**Conclusion:**

This innovative technique allowed a radical excision of forehead DFSP with sufficient healthy margins, thus potentially decreasing tumor recurrence rate. Reconstruction was achieved avoiding microsurgery, skin expanders and large skin grafts. Moreover, all main reconstructive criteria, such as functional and cosmetic tissue characteristics, were completely fulfilled.

## Background

Dermatofibrosarcoma protuberans (DFSP) is a soft tissue neoplasm of intermediate to low-grade malignancy. Although metastasis rarely occurs, DFSP has a locally aggressive behavior with a high recurrence rate [1]. DFSP represents 1% of all soft tissue sarcomas and less than 1% of all head and neck malignancies.

The pathologic characteristics of DFSP were first described by Darier and Ferrand in 1924, [2] with the term originally used by Hoffman in 1925 [3]. The tumor arises from the fibroblastic cells of the dermis and invades deeper subcutaneous tissue. The typical clinical history of DFSP consists of a very slow-growing tumor. It may start as a small asymptomatic white to red papule. Mostly, the tumor is mobile upon palpation but fixation to deeper structures such as fascia, muscle and bone can occur as the tumor progresses. Histologic findings of this neoplasm are represented by spindle cells with a radial arrangement forming a storiform pattern. From this central hub of neoplastic-fibrous tissue, the tumor tends to infiltrate neighboring structures through a radial spreading of up to 3 cm from the primary lesion. Histologic diagnosis can be confirmed by immunohistochemical staining for CD34 [1,4]. In approximately 10% of cases, DFSP may show focal fibrosarcomatous features (DFSP-FS variant). A higher incidence of local relapse and distance metastasis is characteristic of this more aggressive variant.

The characteristic infiltration of surrounding tissues seen with DFSP represents a surgical challenge, since failure of complete excision leads to local recurrence. In the head and neck area, resection involving a 3 cm margin of healthy tissue can be difficult because of functional and cosmetic considerations [5,6]. In this report, we describe our experience at the National Cancer Institute of Naples with two patients who presented with DFSP of the forehead. Both underwent radical excision and reconstruction with an innovative single local wide scalp flap for complete repair of two-thirds of the forehead.

## Case presentation

The novel surgical reconstruction technique described here was performed on two male patients. The first patient had DFSP on the left side of his forehead, while the second presented with a grafted area occupying the whole right half of his forehead, a consequence of previous failed attempts at radical resection of the tumor.

### Surgical technique

The tumor is included in a pentagon-shaped en bloc tissue resection, from skin to the periosteum layer. After excision of the lesion encompassing a 3 cm margin of healthy tissue, reconstruction is initiated. Skin incision is extended through the contralateral eyebrow line, joining the hairline above the auricle, and then continued on over the occipital region.

The scalp flap blood supply is provided by three main vessels: the superficial temporal artery (STa), the occipital artery (Oa) and the posterior auricular artery (PAa), all arising from the external carotid artery, homolateral to the defect area [7]. During the scalp flap dissection, STa, Oa and PAa contralateral to the tumor are detected and ligated. Flap dissection is then carried forward up to the ipsilateral Oa, carefully sparing this vessel. A helpful technique to avoid Oa damage is to place a skin marker at the emerging point of the vessel into the scalp during planning [8]. The Oa arises from the posterior aspect of the external carotid artery, runs deep under the sternocleidomastoideus and splenius capitis muscles, pierces the fascia at the point of connection between the cranial attachment of the trapezius with the sternocleidomastoideus, and runs in the superficial fascia of the scalp. At this point, the dissection continues raising the whole scalp flap from the bone layer and insetting it with rotational movement into the surgical defect, thus completely covering the forehead half to be reconstructed.

### Case 1

**Figure 1:**
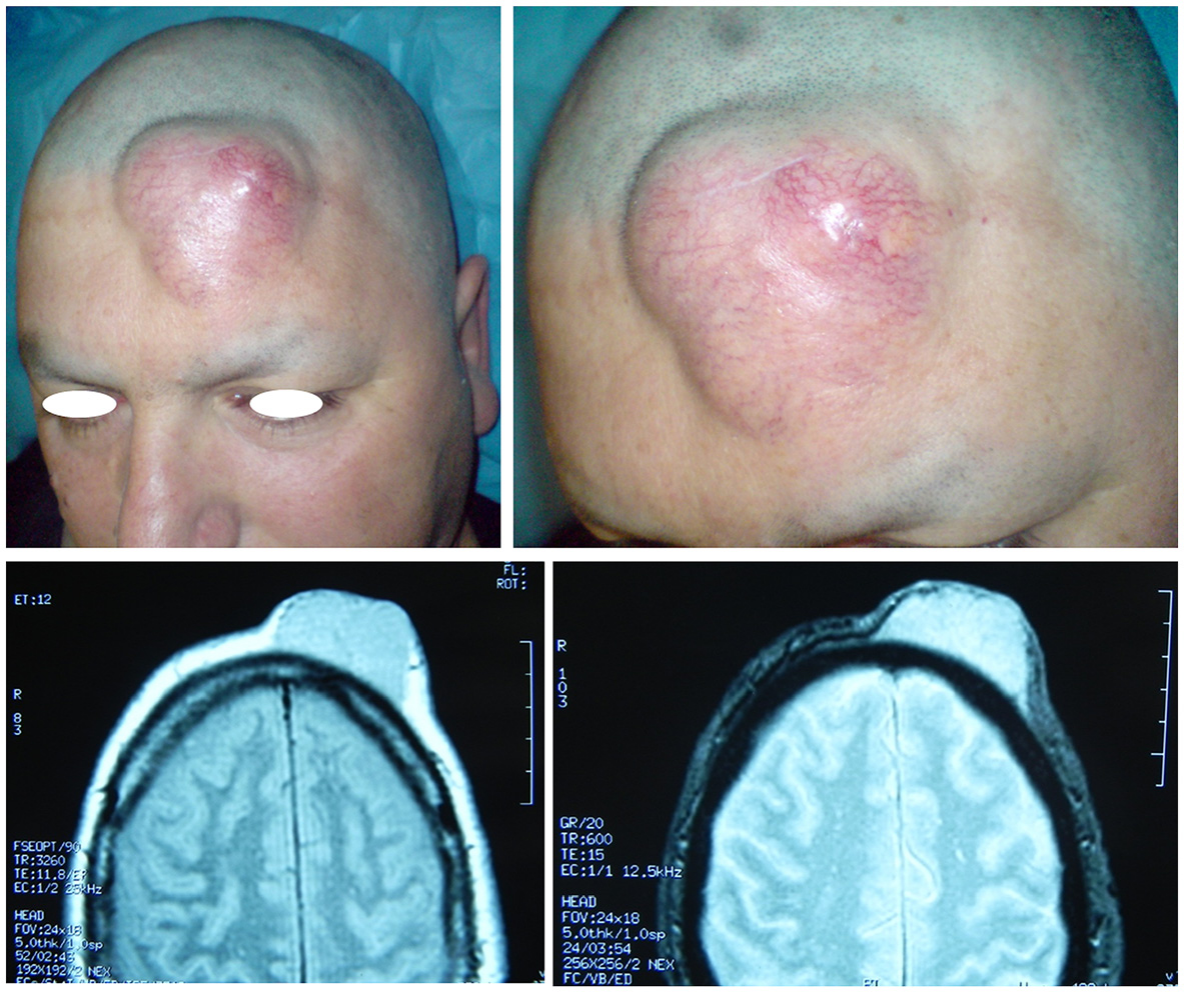
**(Top) Left side of forehead almost completely occupied by DFSP. (Bottom)** Nuclear magnetic resonance aspect of the solid mass demonstrating absence of bone layer infiltration.

**Figure 2:**
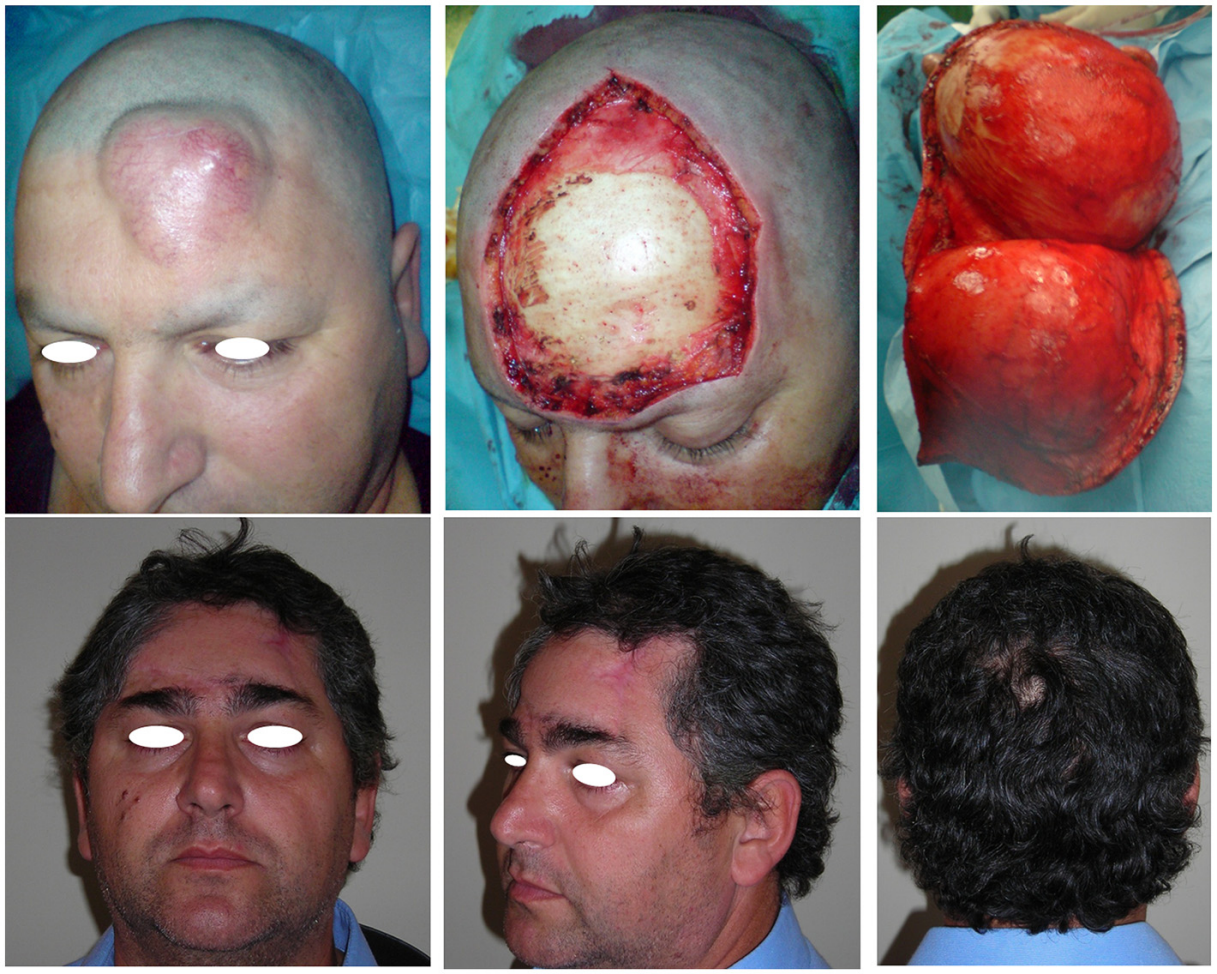
**(Top) After surgical resection with 3 cm margin of healthy tissue.** Scalp flap is completely raised preserving the ‘three arteries pedicle’ homolateral to the tumor. **(Bottom)** Healed flap at one-year follow-up with no asymmetry between right and left eyebrows.

A 52-year-old Caucasian man presented in our outpatient clinic complaining of a swelling on the left side of his forehead that had progressively enlarged over the previous 10 years (Figure 1 (Top)). Examination revealed a subcutaneous 6 × 6 cm solid mass covered by erythematous skin. Ultrasound exploration demonstrated a patchy, solid tumefaction. Nuclear magnetic resonance (NMR) showed a bulky mass occupying the soft tissue area without infiltration of the underlying bone structures, with signal enhancement after contrast medium injection (Figure 1 (Bottom)). Radiographic examination of the chest was negative for disease. Tru-Cut (Baxter Healthcare, Valencia, CA, USA) needle biopsy was performed and the diagnosis of DFSP established. The patient underwent 3 cm-wide surgical resection with bone layer sparing. The operative specimen showed a 10 × 8 cm skin and subcutaneous paddle centered by a solid mass infiltrating the ipodermal layer. The histological report described a mesenchymal neoplasm with spindle cells and storiform pattern. Necrosis was absent with few mitosis in the deeper layer. Immunohistochemical staining demonstrated S100-, CD99-, Bcl2-, CKpan-, Mart1-, PDGFR-B + and CD34 strongly positive. The defect was repaired with a single rotation/advancement forehead scalp flap plus a small skin graft of 3 × 1 cm on the lateral right eyebrow to avoid asymmetries (Figure 2 (Top)). The postoperative course was normal. No surgical complications were observed and the patient remained disease-free at follow-up after 18 months (Figure 2 (Bottom)).

### Case 2

**Figure 3:**
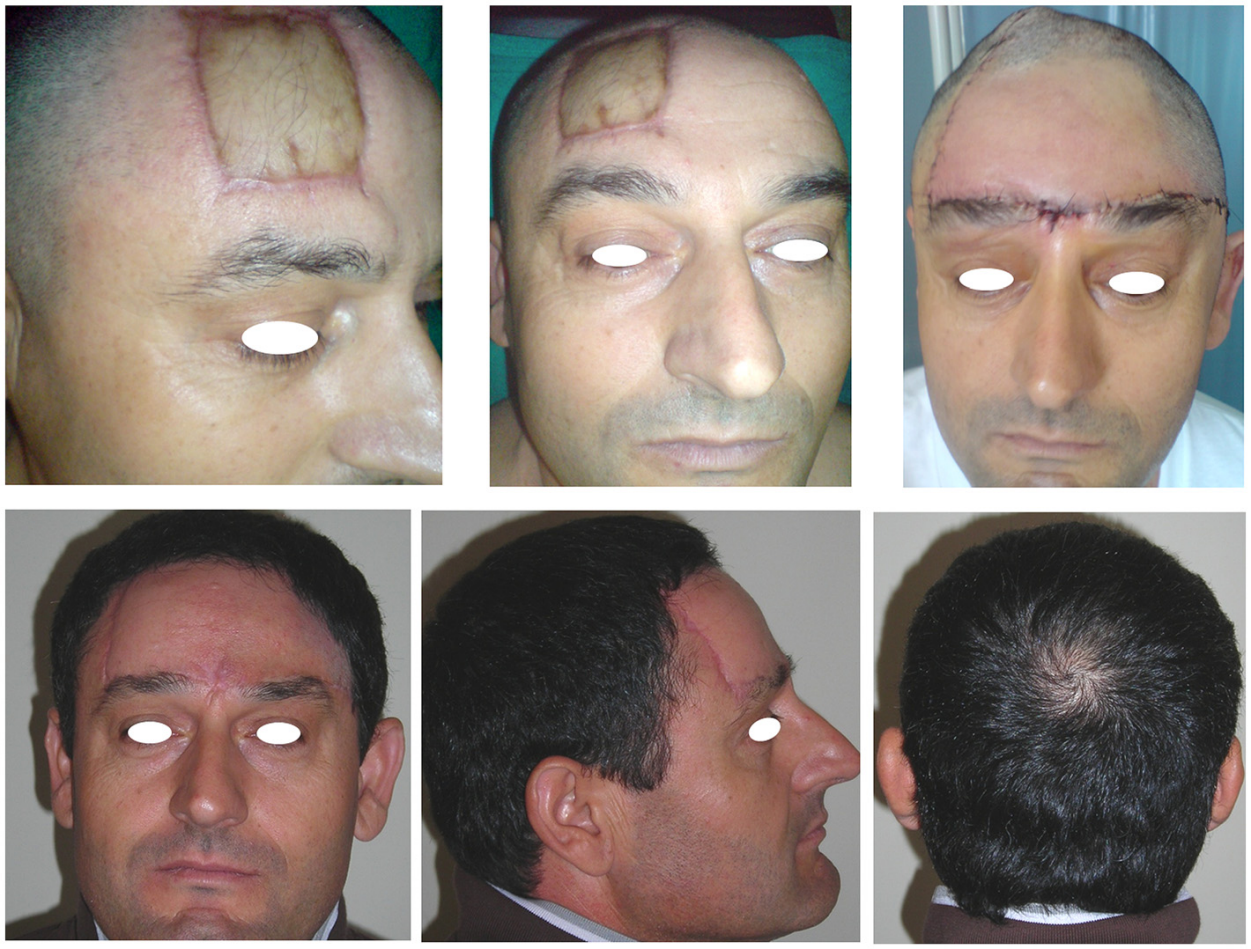
**(Top left) and (Top center) Right side of forehead showing a skin graft following an incomplete excision of DFSP. (Top right)** Reconstruction with a single forehead scalp rotational flap. **(Bottom)** Healed flap at one-year follow-up.

This was a 45-year-old Caucasian man with a 14-year history of a frontal skin solid mass with infiltration of the hypodermal layer and lateral margins. The tumor had been excised in a different hospital following a diagnosis of DFSP, but two local relapses had subsequently occurred. A skin graft on the right side of the forehead was the result of previous failed attempts at radical resection (Figure 3 (Top left) and (Top center)). The patient underwent a wide local excision, including the underlying periosteum, with a 3 cm margin. Reconstruction was performed with a forehead-scalp rotational flap, avoiding any additional skin graft (Figure 3 (Top right)). Histologic analysis revealed recurrent disease in the reticular dermis layer. The patient was recurrence-free at the 18-month follow-up (Figure 3 (Bottom)).

## Discussion

Since elective treatment of DFSP involves wide surgical resection of the lesion with 2 to 3 cm of surrounding healthy tissue, occurrences in the head and neck are challenging because of the risk of cosmetic disfigurement and functional impairment [1,9-11]. In recent years, cervicofacial reconstruction has provided various and valid surgical solutions [12]. However, microsurgical reconstruction is often burdened with high rates of partial or total flap failure, bulky and mismatching skin paddle, and a lengthy recovery period with high associated economic costs [5]. Moreover, facial DFSP treatment is a rare and poorly described topic.

Half forehead reconstruction following tumor resection requires considerable experience and extensive knowledge of anatomical details. Most common reconstructive methods are performed by means of tissue expanders and microvascular free flaps. We considered the former technique not ideal because of the mandatory skin expansion time required to provide defect closure. Additionally, expander placement entails a four- to six-month period during which patients bear these clearly observable devices, and involves elaborate surgical planning. Microvascular free flaps may have been a better option but also have several disadvantages.

H-plasty procedure is a bilateral advancement flap for closure of small- to medium-sized forehead defects that cannot be closed primarily [13]. In both cases presented here, tumor size plus the required healthy tissue margins left a half forehead defect too large to be reconstructed with a simple H-plasty technique.

The main clinical characteristic of DFSP is the high local recurrence rate, which is probably related to the presence of neoplastic tissue with radial spreading up to 3 cm from the site of the primary lesion. The cases reported here presented with clinical histories of 10 and 14 years respectively. Previous attempts of surgical tumor removal were unsuccessful. Given their respective histories, a surgical approach that involved closer healthy tissue margins would involve a risk of relapse too high for these patients.

The novel reconstructive method that we have developed uses a total scalp and remaining contralateral forehead flap. The scalp flap is planned as a single wide flap. This differs from the previously described Orticochea flap since the Orticochea procedure was based on three different flaps [14]. Moreover, the Orticochea technique is indicated for scalp defects, but is not the treatment of choice for forehead reconstruction. The scalp flap described by Converse in the 1940s is a procedure for nasal reconstruction, based on a forehead flap [15]. We consider the single rotational scalp flap that we describe as different since it is a scalp flap planned for forehead reconstruction.

Scalp blood supply is based on ten main arteries, five on each side, with two arising from the internal carotid artery (supratrochlear and supraorbital artery) and three arising from the external carotid artery (STa, Oa and PAa) [7]. In performing this surgical procedure, the blood flow from seven of these arteries is interrupted: the four vessels coming from the internal carotid artery are all sectioned during tumor resection and forehead skin incision through the eyebrow, and the three vessels coming from the external carotid artery contralateral to the tumor are sectioned in raising the scalp flap for reconstruction purposes. Thus, the three remaining arteries arising from the external carotid artery homolateral to the excised DFSP (STa, Oa and PAa) have to supply the whole dissected scalp.

**Figure 4:**
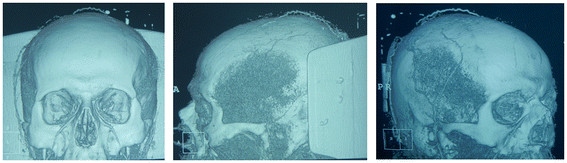
**(Case 2). Six months’ post-operative computed tomography (CT) angiography.** This demonstrates the absence of flow in both the supratrochlear and supraorbital arteries, and in the superficial temporal, occipital and posterior auricular arteries on the contralateral side to the tumor. Blood flow is detectable in the superficial temporal, occipital and posterior auricular arteries homolateral to the tumor.

Six months’ post-surgery, computed tomography (CT) angiography demonstrated the presence of blood flow from the three vessels (STa, Oa and PAa) homolateral to the tumor. These three arteries rise in a tortuous course in the superficial fascia of the scalp, and then divide into numerous branches, which come up to the vertex of the skull and anastomose each other (Figure 4). For this reason, three of the ten arteries of the total scalp blood supply give enough nourishment to the entire scalp and forehead tissue.

The temporal branch of the facial nerve traverses inside the deep layers of the temporoparietal fascia and the superficial musculoaponeurotic system along the zygomatic arch. It supplies innervation to the corrugator supercilii, frontalis and orbicularis oculi muscles [16]. Surgical injury to this nerve leads to inability to elevate the eyebrows and brow ptosis. In both presented cases, tumor radical resection needed complete soft tissue removal from skin to bone layer excluded. An ipsilateral frontalis and corrugator supercilii muscle deficit should be a certain consequence. Nevertheless, there was no asymmetric appearance of the forehead in either case and no brow ptosis occurred. Careful pre-operative planning of the flap played an important role in avoiding these complications.

In the single rotational scalp flap planning for half forehead reconstruction, skin incisions are carried out just over the eyebrow joining the contralateral hairline above the auricle, toward the occipital region. Thus, the final wound lies laterally to the exterior margin of the eyebrow, ipsilateral to the defect. Hair-bearing skin is moved forward 1 to 2 cm within the final reconstructed forehead, contralateral to the defect. Neither patient complained about this aspect of the procedure. Nevertheless, laser hair removal could be provided in the future if considered beneficial.

## Conclusion

In our opinion, this innovative technique allows a radical excision of forehead DFSP with sufficient healthy resection margins, thus potentially decreasing tumor recurrence rate. Microsurgery, use of skin expanders and large skin grafts were avoided. Additionally, all main reconstructive criteria, such as functional and cosmetic tissue characteristics, were completely fulfilled.

## Consent

Written informed consent was obtained from patients for publication of their clinical details and accompanying images. A copy of the written consent is available for review by the Editor-in-Chief of this journal.

## Abbreviations

DFSP = Dermatofibrosarcoma protuberans; FS = Fibrosarcomatous; NMR = Nuclear magnetic resonance; Oa = Occipital artery; PAa = Posterior auricular artery; STa = Superficial temporal artery.

## Competing interests

The authors declare that they have no competing interest. There is no external source of funding involved in the submitted article.

## Authors’ contributions

SM conceived the study and realized the technique. GDM drafted the manuscript, helped to conceive the study and carried out the literature research. UM helped in the preparation of the manuscript. MGC carried out literature review. CC carried out literature review and helped in management of the patients. All authors read and approved the final manuscript.
